# Prospects for Psychological Support in Interplanetary Expeditions

**DOI:** 10.3389/fphys.2021.750414

**Published:** 2021-11-03

**Authors:** Vadim Gushin, Oleg Ryumin, Olga Karpova, Ivan Rozanov, Dmitry Shved, Anna Yusupova

**Affiliations:** ^1^Russian Federation State Scientific Center - Institute of Biomedical Problems of the Russian Academy of Sciences, Moscow, Russia; ^2^Moscow Aviation Institute, National Research University, Moscow, Russia

**Keywords:** manned space flights, interplanetary space flights, psychological factors of interplanetary flights, psychological support, virtual reality

## Abstract

The article gives an overview of Russian experience in psychological support for orbital space flights. It describes procedures that currently exist and may possibly be used in upcoming manned interplanetary flights. The article also considers psychological unfavorable factors of autonomous interplanetary flights, as well as countermeasures, including promising methods of psychological support.

## Introduction

The existing Russian space crews’ psychological support has shown its efficiency in support for long-term space flights. The most important means of the current support system are based on the possibility of continuous communication between Earth and spacecraft by e-mails and IP-telephony, as well as regular cargo deliveries from Earth. However, in interplanetary flights, there will be an increasing delay in communication with the crew. This will lead to the loss of the direct “live” dialogue between Earth and astronauts, which is now usual for the crews, and the impossibility of prompt medical and psychological assistance. Therefore, in case of emergencies that require immediate solutions, crews will have to rely on their own decisions. It will also be difficult to maintain social contacts with families during interplanetary flight. Also, resupplies of food, photos and videos, books, surprises, etc. that help to break sensory deprivation and monotony with cargo ships would also become unavailable. Several authors, describing problems of interplanetary flights, mentioned the phenomenon of “disappearing Earth.” The absence of visual contact with Earth could be expressed in the feeling of connection loss with the home planet, its culture, and society that could cause subsequent depression and motivation decline in astronauts. In the orbital flights, Earth observations are the most preferable way of spending free time, thus being the main source of changeable external stimuli. All these news unfavorable factors cause a need to form new approaches to psychological support for the interplanetary space crews, based on the detailed consideration of these problems and limitations of interplanetary flights.

## Psychological Support in Orbital Space Flights

Medical and psychological support in Soviet/Russian long-term flights revealed a number of psychological problems in space crews performing complex activities under conditions of prolonged exposure to microgravity and confinement and a constant threat to life and health. These include emotional and sleep disorders, decreased performance, psychological tension inside the crew as well as in relations with the Mission Control Center (MCC), etc. Russian psychiatrists and psychologists, in particular (Myasnikov, 1988) and his team, attribute the appearance of these phenomena to development of asthenization of central nervous system caused by sensory deprivation (a significant decrease in external sensory inflow), as well as the absence of external time indicators (desynchronosis), and monotony caused by prolonged stay in the small chambers with artificial environment formed by the life support systems of space stations. Another unfavorable factor was considered by Novikov (1970, 1981) is social deprivation, narrowing of the usual social contacts, and the imposed communication with Mission control (MCC) specialists.

To deal with these problems, a team led by O.G. Gazenko and O.P. Kozerenko developed a system of measures for psychological correction and support of space crews (Kozerenko et al., 2001). This system is based on experience collected within the frame of practice of medical and psychological support for early Salyut and MIR orbital stations. This system is also based on the results of numerous terrestrial extended chamber studies. The main principle of this support system is to counterbalance each unfavorable factors’ group by corresponding preventive measures.

The first group of unfavorable psychological factors includes sensory deprivation and monotony. These factors lead to the so-called sensory “hunger,” that appears in a decrease in tone and sensitivity of the central nervous system, deterioration of cognitive functions, in particular, attention and memory, as well as in growing stereotyping of actions, appearing anxiety and irritability. To prevent this, a group of psychological support measures is used to restore the usual sensory input (Myasnikov and Stepanova, 2002).

Crews are systematically provided with external data sources, at the initial stage during the “Salyut” era – with books, magazines, and music records. On MIR and ISS, the crew is provided with digital content storages, containing video and audio information that was constantly updated by the support service on crew demands. The content provided to each cosmonaut is individual, based on his personal preferences. The reduced muscular proprioceptive flow caused by microgravity is compensated by a large volume of regular physical exercises, so the crewmembers always mention that it makes positive effect on their mental state.

The second group of unfavorable psychological factors is caused by social deprivation. In flight, the communication of the crew is an element of their professional activity. While the volume of compulsory professional communication increases dramatically from the very first days of flight, there is a significant decrease in the number and duration of contacts with the close social circle. So, each person on orbit is deprived from the usual social support from family and friends (Novikov, 1970). As a result, we observe growing feelings of loneliness and detachment from the family so sometimes it has an adverse effect on astronaut’s family life (Suedfeld, 2012). Thereby, a second focus of psychological support measures is to maintain for each crewmember in flight the usual circle of contacts and social bounds. Psychological support measures dealing with factors listed above include informal communication between cosmonauts and MCC management, private communications with family and friends, and private conferences with psychologists, proposed by NASA and used in Russian system of support. Additionally, psychological support team organizes video and audio calls between cosmonauts and celebrities, such as famous artists and actors, politicians, religious leaders, and athletes. These interactions also help to keep crewmembers motivated.

The existing approach for psychological support confirmed its effectiveness in numerous orbital space Missions of various duration (Myasnikov, 1988). Its utilization started on Salyut orbital stations within the frame of 17 long-term expeditions and 15 visiting crews (10 of these were international). The same approach was then used for 28 expeditions that worked at the Mir orbital station, including nine international ones, and for all Russian crewmembers on ISS.

Russian system of psychological support is now internationally approved by Space agencies. One of the prominent NASA psychologists, Albert Holland, confirmed that American inflight psychological support is based on the principles, established by Russian system (Burrough, 1999). Common approach to psychological support allows NASA, ESA, and Institute for Biomedical problems RAS to test together some new means of psychological support described below (e.g., virtual reality and growing plants) in the international series of isolation studies SIRIUS.

## Psychological Factors of Interplanetary Flights

Interplanetary space flights could possibly be accompanied by technical difficulties, along with a longer mission duration without any possibilities for resupply or crew immediate evacuation or change (e.g., due to a technical malfunction or crewmember illness). In addition, a number of new psychological factors that might become typical for interplanetary flights (e.g., communication delay) should be considered. These factors, which possible impact is described below, could cause serious limitations in utilization of the current psychological support system and should be taken into account when arranging psychological support for long-term human interplanetary flights (Grigoriev et al., 2012).

### Communication Delays and No Additional Deliveries

In interplanetary flights, an increasing delay in communication with crews is inevitable. In a flight to Mars, the delay can take up to 24min (in each direction). Communication delays would lead to the impossibility of prompt assistance, advice, and additional briefing from Mission Control. In case of critical situations requiring an urgent solution, the crew would have to rely on their own decision-making – without external advice and expert opinion. No psychological support in live audio contacts with Mission controllers will be available. This would increase the autonomy of the crew.

Results of chamber experiments such as Mars-500 and SIRIUS show that the communication delay affects the communication of the crew both with the Mission Control and with the Earth in a significant and, basically, negative manner (Ushakov et al., 2014; Gushin et al., 2020).

In an interplanetary flight, delivery with transport ships of food, photo, and video materials, books, and surprises would also be unavailable. Thus, deliverables can be used to break monotony and sensory deprivation. It complicates the task of a constant remote monitoring, correction of the psychophysiological state and performance, and individualized compensation of sensory deficiency.

### “Disappearing Earth” Phenomenon (the “Break-Off”)

(Kanas and Manzey, 2008; Kanas, 2015), being the authors of this term, initially associated this phenomenon’s development with the loss of visual contact with the planet. They suggested that when there would be no usual view of home planet’s surface in the spacecraft window but a motionless picture of an endless starry sky instead, this could become a stressor in an interplanetary flight and could be regarded as a loss of a psychological “anchor.” Currently, the “Disappearing Earth” phenomenon is described as a progressive rise of homesickness, emotional depression, apathy, and melancholy coming with increasing distance from the home planet and physical autonomy.

Both in the scientific literature and in cosmonauts’ memoirs, the psychological impact of looking at Earth from the orbit has been described as one of the main pleasures in flight. Multiple reports show that Earth’s observation has always been one of the favorite ways of spending free time of astronauts (Johnson, 2010). Cosmonauts and astronauts of different generations (Ritsher et al., 2007) tell that they were deeply influenced by the views of the Earth and became more responsible for ecological issues and for interpersonal relations. These are the main drivers of the so-called salutogenesis phenomenon when individuals who can adapt to the demands of an inhospitable or extreme environment can derive benefit from their experiences (Antonovsky, 1987). Under conditions of a reduced sensory influx, terrestrial landscapes make it possible to compensate sensory deprivation and monotony effects. It is highly likely that in a reduced sensory influx, the colorful and diverse terrestrial landscapes partly compensate the effects of sensory deprivation and monotony (White, 1987, 2021) that space flights form a special attitude (overview effect) toward the planet in astronauts and cosmonauts, a feeling of its fragile beauty, a deep spiritual connection with life on Earth. Same evidence was presented by Kanas and Manzey (2008).

In the long-term isolation Mars-500 study, it was found that prolonged lack of visual contact with Earth may also cause decrease in overall crew activity and motivation. In Mars-500, crew’s behavior progressively became more independent of the MCC recommendations and even aggressive. So, subjects’ “autonomy” together with communication delay led to a general decrease in the crew’s need for assistance and guidance from the ground services – but still there was a growing need for psychological support (Feichtinger et al., 2012; Gushin et al., 2012). Also, this phenomenon may be accompanied by the so-called “groupthink” (Kanas et al., 2009).

### Other Factors

Other significant adverse psychogenic factors of interplanetary flights that should be also considered are their long duration, limited, or complete absence of the possibility of early mission termination and substitution of the crew member (e.g., due to force majeure such as equipment failures, meteorite collision, and crewmember’s illness). It is necessary to note the important role of activities requiring independent task setting and the solution searching, as well as high requirements for the crew’s ability to make prompt decisions in an *ad hoc* environment (Kanas and Manzey, 2008; Kanas, 2015). These factors are likely to have a negative impact on the psychological status and therefore should be taken into account when building a new system of psychological support.

## Limitations of Existing Methods of Psychological Support in Interplanetary Flights

Communication delays and impossibility of additional deliveries impose restrictions on usual psychological support measures and narrow the quick help opportunities for crewmembers in interplanetary flight. Due to communication delays in crew – Earth communication, such countermeasures for social deprivation as informal talks with ground personnel, family, friends, and colleagues, as well as celebrities, would be significantly limited. Communication delays would certainly reduce the possibility of immediate negative emotions drainage (transfer) through private psychological sessions with psychiatrists, informal communication with ground personnel, described by Beregovoy et al. (1993) and Kanas et al. (2008).

Because of delays, interplanetary mission crews would receive news from Earth later than usual. It is important to mention that the positive impact of a prompt news flow is not only in maintaining communication about events on Earth, but also in positive feedback about crewmembers’ activity in mass media. One of the famous examples of the positive impact of news from Earth on crew morale is the first Soyuz when newspapers delivered by the new coming crew caused a positive emotional response in the onboard crew (Kamanin, 1969). Cargo ships bring supplies with spare parts and consumables, that gives a crew a sense of protection, support, and brings certain confidence (Savinykh, 2017). So, cosmonauts, as well as participants of long-term confinement experiments, note improvements in mood, activity, interest, and motivation to continue their work after receiving personal parcels in cargo ships (Bojko, 1975). In an interplanetary flight, the parcels, that might have created a sense of connection with the Earth (Ryumin, 1987), would be inaccessible.

With the absence of resupplies, it would also be difficult to maintain food diversity. In sensory deprivation and monotony, food rations should not only compensate the energy loss, but satisfy the aesthetic and cultural requests of the crew (Lane and Feeback, 2002). In Mars-500 experiment, one of the crewmembers experienced serious psychological difficulties because of the standard food ratio’s composition. These difficulties vanished after national cuisine elements and spices were added to the facility (Ushakov et al., 2014).

## New Solutions and New Technologies for Psychological Support in Interplanetary Flights

Even though a number of support measures listed above would still be available in interplanetary flights, we may assume that these standard measures could be not enough to countermeasure the complexity of new unfavorable psychological factors of interplanetary flights. That is why space psychologists are developing new means of psychological support for flights beyond Earth orbit.

### Virtual Reality Technologies

In our opinion, one of the most promising new psychological support methods could be based on virtual reality (VR) technologies. VR is capable to create an interactive world of diverse and changing visual images that make deep sensory immersion possible (Baños et al., 2000). We suggest that in an autonomous interplanetary flight, when live communication would be lost because of the communication delay and Earth would be out of sight, the VR technology may somehow prevent negative effects of sensory deprivation and monotony, as well as homesickness and loss of memories about usual life on Earth. VR may serve as a temporary replacement for the lost terrestrial reality and compensate for insufficient afferentation by creating an additional, artificial afferentation source (Rozanov, 2020). Thus, VR methods may become an effective countermeasure to such negative factors as deficit of sensory influx (while creating an artificial visual afferentation), crowding (while creating a virtual personal space), and monotony (an interactive VR method might help structure leisure time). Experience of virtual worlds that are creating positive emotions may contribute to optimization of crewmembers’ psychophysiological state.

General tasks of VR for psychological support may be seen as:

- Restoration of the Earth’s nature and usual life images, that may get partly lost from memory during spaceflight isolation as a countermeasure to the “Disappearing Earth” effects. We could use VR reproducing views of certain Earth regions using volumetric video or 3D computer models.
- VR as an organized form of leisure (countermeasure to monotony and asthenization).
- Countermeasure to the lack of physical personal space by creating a virtual personal space. Crewmembers would be able to create their own virtual “homes.” It might be possible to change its design, repair it, make equipment, and do gardening. Virtual reality means may also include virtual “windows” (screens), an interactive spacecraft interior solution. These windows can create an illusion of a landscape seen behind the chamber wall. This view may be dynamic, that is, changing over time (daily change of lighting, of seasons, and of landscapes). Virtual windows may also include cameras to track the movement of user’s sight. The image in the virtual window is distorted in accordance with the movement of the user’s head and create an illusion of volumetric space behind this window. This technology has already been used in commercial products and is being tested for use in psychotherapy (Burdea, 2002). Virtual windows may be used not only for recreation, but also as a daily routine pacemaker, changing luminosity, and imitating Earth’s daily rhythm.

**Figure 1:**
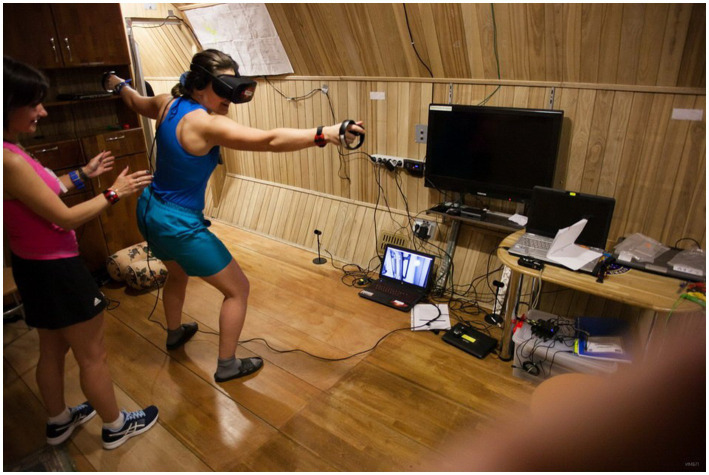
A member of the Roscosmos Cosmonaut Corps, A. Kikina, uses VR for recreation during the SIRIUS-17 experiment. Copyright (2017) by the Institute of Biomedical Problems, Russian Academy of Sciences reprinted and adapted with permission.

In SIRIUS-17 and SIRIUS-19 chamber studies, organized by IBMP in cooperation with NASA and simulating extended interplanetary Mission of the international crews, VR was tested for the first time together with standard psychological support procedures. In these experiments, positive effect of VR’s utilization on crewmembers’ emotional state was observed (Figure 1). Members of Russian astronaut core, participating in the studies, regarded VR as a useful tool to brake monotony, boredom, and to train professional skills.

**Figure 2:**
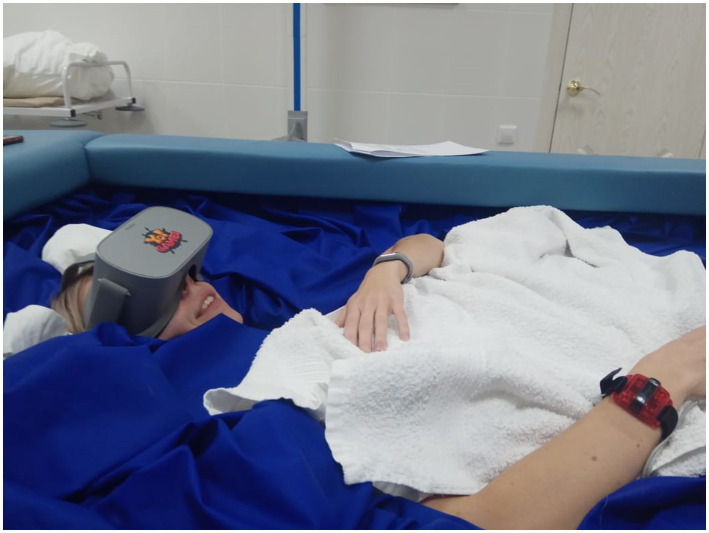
Using a VR as a psychological support tool during «dry» immersion. Copyright by Ivan A. Rozanov, October 2020.

**Figure 3:**
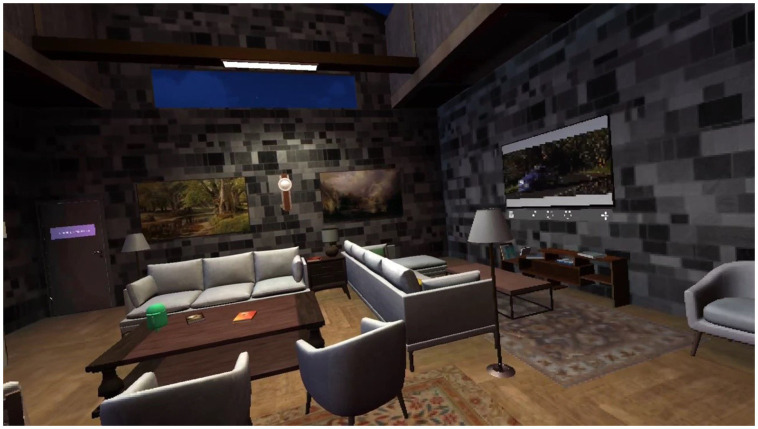
A virtual personal space in the VR environment, developed by the IBMP specialists (together with the AI Health LCC) for psychological support in conditions of crowding and lack of privacy. Copyright by AI Health LCC and Ivan A. Rozanov, March 2021.

VR for psychological support was also tested in one of IBMP’s Dry Immersion experiments (Figure 2). The model of Dry Immersion artificially creates conditions similar to zero gravity *via* floatation in a deep bath using elastic fabric separating the subject’s body from water. Thus, the Dry Immersion reproduces three effects of weightlessness: physical inactivity, support withdrawal, and elimination of the vertical vascular gradient (Tomilovskaya et al., 2019). In conditions of Dry Immersion, VR utilization not only caused relaxing, emotion-balancing effect of VR on the subjects, feeling back pain and irritability, but also had positive effect on cognitive area (Rozanov et al., 2021; Nosenkova, 2021; Figures 3). Also, VR technologies for psychological support were tested by NASA in analogous conditions (South Pole and Hawaii research stations: Anderson et al., 2016).

Certainly, these data need further confirmation and more statistically significant results are necessary to make reliable conclusions. With all the promise of VR technology, its use in psychotherapeutic practice sometimes causes ambiguous effects, both psychophysiological (motion sickness) and psychological. This requires a careful assessment of possible psychophysiological effects that may arise in recipients using VR for psychological support. VR programs should be subjected to the same “psychological safety” requirements as media library content. They should not cause negative mental shifts or remind crewmembers of traumatic events.

### Voice Assistants

Voice assistants, robots, and virtual assistants may serve for psychological support to overcome social deprivation and the impossibility of direct communication with family members and friends caused by communication delays.

Social robotics [an interdisciplinary area robotic systems development that are intended for social interaction with people (Kemke, 2007)] are now used in various fields, such as social protection, psychological rehabilitation, and others. Social robots for crewmembers’ psychological support under loneliness and isolation may possibly use the experience already acquired in robotics for hospital patients and elderly people. Social robots may serve in possible long-term autonomous and interplanetary space flights and in creation of habitable stations on space objects (Sorokin et al., 2016).

Voice monitoring technologies and interaction with automated systems through speech synthesis may also serve for psychological support. Voice assistants might be able to compensate the lack of audial communication in a small team, especially when accompanied by a delay or even absence of communication with the Earth. Voice assistants may not only help cosmonauts to interact with equipment and provide necessary technical data, but may also become a certain kind of companion capable of non-professional communication with crewmembers (Gough et al., 2006).

Therefore, the functions of voice assistants may be as follows: compensation of lack of information in autonomous conditions, organization of leisure time (presentation of news, references, and entertainment information, interactive games with voice support), and psychotherapy based on active listening. It may also become possible to assess crewmembers’ emotional state by recognition of facial expressions and analysis of acoustic parameters of their speech and to provide an adequate psychological support in accordance with subject’s reactions detected.

### Spacecraft Greenhouse

**Figure 4:**
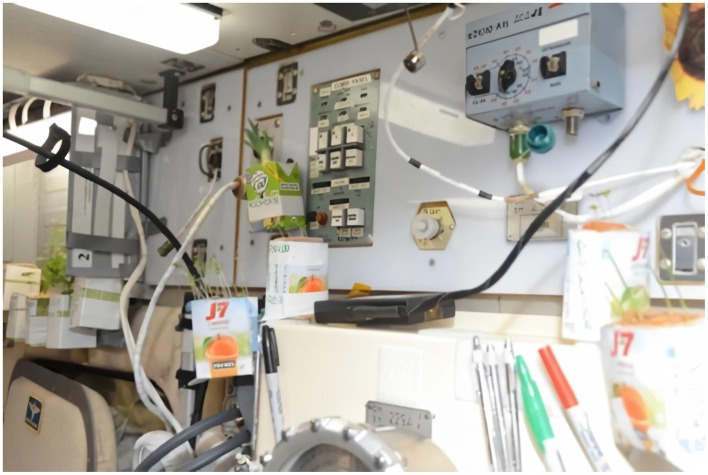
Improvised “houseplants” on board the ISS, grown by the cosmonauts for additional psychological support.

**Figure 5:**
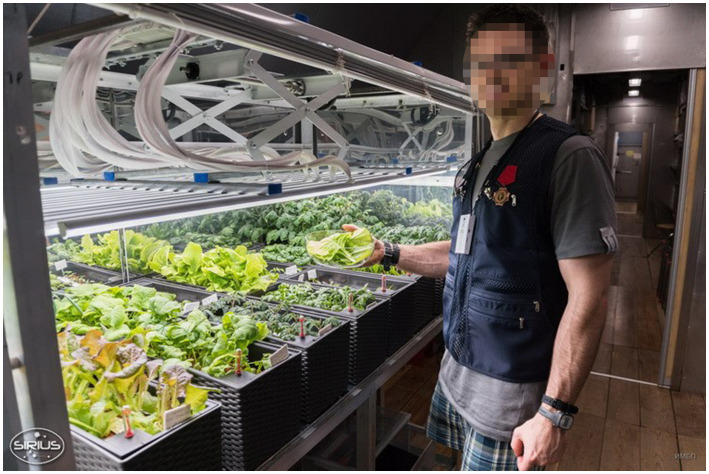
A participant of the SIRIUS-19 with the onboard greenhouse. Copyright (2019) by the Institute of Biomedical Problems, Russian Academy of Sciences reprinted and adapted with permission.

A greenhouse on board is not only an element of a closed life support system and a source of food that adds variety to the food intake. From several spaceflights anecdotal information and studies in space simulations, we got evidence that onboard greenhouses are effective for psychological relaxation. Both in space flights (mini greenhouses were installed on board) and in isolation experiments, there was a positive effect of greenhouses on crews’ emotional state (Figures 4, 5). The greenery grown on board contributes to the restoration of the image of the Earth. Caring for plants is regarded by crewmembers as a form of leisure that adds variety to the flight routine. This may be important in long-term missions, in a visually impoverished interplanetary space, and during possible colonization of other planets, with no local flora and fauna (Bates et al., 2009; Gushchin et al., 2014).

In Mars-500, one of IMBP’s long-term confinement experiments that simulated an interplanetary expedition, the influence of plants on crewmembers’ emotional state was found (Gushchin et al., 2014). The location of greenhouse near recreational areas, as well crewmembers’ involvement in plant care, caused the positive psychological effect. The crew preferred to grow large, brightly colored flowers that did not require much maintenance. Also, the research results showed that the choice of edible plants to be grown should consider crewmembers’ cultural food preferences.

Another discussed possibility of psychological support in interplanetary flights and possible settlements is to include animals in closed life support systems. The beneficial psychological effect of laboratory animals taken in flight for experiments has already been described (Sychev et al., 2008).

## Conclusion

A broad view on the existing psychological support system in orbital flights, analysis of ground-based isolation experiments results, and the studies of psychological particularities of interplanetary space flights suggest that the possible future set of psychological support measures needs to be created with taking several crucial issues into account:

1. Support in the interplanetary missions should be based on the available experience of psychological support of orbital flights, but future system should not depend on online audio communication and resupplies from Earth, as it is now.
2. New unfavorable psychological factors of interplanetary missions (autonomy, communication delay, “disappearing Earth phenomenon,” etc.) should be taken into account.
3. Support methods should be autonomous and could be used by crews without any additional advice from Earth, based on the information they have above and needs they experience.

## Author Contributions

All authors listed have made a substantial, direct and intellectual contribution to the work, and approved it for publication.

## Funding

The study was supported by the Ministry of Science and Higher Education of the Russian Federation under the agreement No. 075-1502020-919 from 16.11.2020 about the grant in the form of subsidy from the federal budget to provide government support for the creation and development of a worldclass research center “Pavlov Center for Integrative Physiology to Medicine, High-tech Healthcare and Stress Tolerance Technologies.”

## Conflict of Interest

The authors declare that the research was conducted in the absence of any commercial or financial relationships that could be construed as a potential conflict of interest.

## Publisher’s Note

All claims expressed in this article are solely those of the authors and do not necessarily represent those of their affiliated organizations, or those of the publisher, the editors and the reviewers. Any product that may be evaluated in this article, or claim that may be made by its manufacturer, is not guaranteed or endorsed by the publisher.
